# Prevalence and Association of Elevated Liver Transaminases in Type 2 Diabetes Mellitus Patients in Jeddah, Saudi Arabia

**DOI:** 10.7759/cureus.5166

**Published:** 2019-07-18

**Authors:** Sami H Alzahrani, Mukhtiar Baig, Jamil I Bashawri, Mooataz M Aashi, Faisal K Shaibi, Dalya A Alqarni

**Affiliations:** 1 Family Medicine, King Abdulaziz University, Jeddah, SAU; 2 Medical Education and Simulation, King Abdulaziz University, Jeddah, SAU; 3 Pediatrics, King Abdulaziz University, Jeddah, SAU; 4 Medicine, King Abdulaziz University, Jeddah, SAU

**Keywords:** alt, ast, hba1c, smoking, diabetes mellitus

## Abstract

Background

This study investigated the prevalence and association of liver transaminases in type 2 diabetes mellitus (T2DM) patients at King Abdulaziz University Hospital (KAUH), Jeddah, Saudi Arabia.

Methods

This retrospective, cross-sectional study was carried out on 211 T2DM patients at KAUH in 2017, and the Research Ethics Committee of KAUH approved this study. The data were analyzed on SPSS 21 (IBM Corp., Armonk, NY, US). The association of aspartate aminotransferase (AST) and alanine aminotransferase (ALT) with several risk factors was computed by the chi-square test. The odds ratio with a 95% confidence interval (CI) was also calculated.

Results

The mean age of study participants was 60 ± 13.43 years; 143 (67.8%) were female while 68 (32.2%) were male. Serum AST levels were elevated in 6.16% (10.3% in males, 4.2% in females). Elevated ALT levels were found in 7.58% (11.8% in males, 5.6% in females) (Table 2). The probability of rising AST levels increased with age (OR = 2.59 for patients aged 46-65) and with male gender (OR = 2.65, CI: 0.84-8.12). Additionally, the probability of rising ALT levels increased with male gender (OR = 2.25, CI: 0.80-6.27), low-density lipoproteins (LDL-C) (OR = 2.11, CI: 0.73-6.04), and triglycerides (TG) (OR = 2.08, CI: 0.739-5.87). No statistically noteworthy association was observed between elevated levels of AST and ALT with gender, age, body mass index (BMI), glycated hemoglobin (HbA1c), TG, total cholesterol (TC), LDL-C, and high-density lipoprotein cholesterol (HDL-C) levels, smoking, or hypertension.

Conclusion

Higher ALT and AST levels were found in T2DM patients but with no statistically significant link between elevated levels and gender, age, BMI, HbA1c, TG, TC, HDL-C, LDL-C, smoking, or hypertension.

## Introduction

The liver is a metabolic food factory where, generally, all types of nutrients and, particularly, carbohydrates, proteins, and lipids metabolize. It plays an important role in maintaining blood sugar levels in both a fasting state and a postprandial state. The World Health Organization (WHO) reports that almost 422 million people have type 2 diabetes mellitus (T2DM) globally, and in 2025, that number will be doubled. Furthermore, developing countries will suffer more because of unhealthy diets, sedentary lifestyles, obesity, aging, and high population growth [1]. According to the report from the WHO, the overall prevalence of T2DM in Saudi Arabia is 14.4% (14.7% males and 13.8% females) [2].

Diabetes causes lipid disorders and, consequently, long-term complications and injury to many organs of the body, including the liver [3-4]. Moreover, T2DM is considered one of the causes of liver diseases such as nonalcoholic steatohepatitis (NASH), nonalcoholic fatty liver disease (NAFLD), cirrhosis, and, eventually, hepatocellular carcinoma [5].

Liver disease comprises substantial comorbidity in T2DM [6]. A few studies have reported that liver injury biomarkers can independently forecast the possibility of deranged fasting sugar, T2DM, and metabolic syndrome (MS) [7-8]. Diabetes can impair liver and heart muscle cells, which, in turn, may influence levels of serum alanine aminotransferase (ALT), aspartate aminotransferase (AST), alkaline phosphatase (ALP) and creatine kinase (CK) [9].

The literature indicates a comparatively high prevalence of abnormal serum liver enzymes, specifically ALT and AST, in diabetic patients [6,10-11]. However, Ahmadi found no significant increase in ALT or AST levels [12].

Thus, in spite of the abundant literature, the range of elevated ALT and AST in T2DM patients is still indistinct. It is imperative to investigate these liver enzymes in diabetic patients because the literature indicates that T2DM accompanied by the derangement of liver enzymes establishes an augmented risk for cardiovascular disease [13-14] and kidney disease [15].

In the Saudi population, the relationship between liver enzymes and T2DM has not been extensively studied. Further, studies about the magnitude of elevated liver transaminases in the Saudi Arabian population are lacking. However, a few studies have been done in the Middle East that tackled this topic [16-17]. Therefore, our study aimed to investigate the prevalence and association of liver transaminases among patients with T2DM at King Abdulaziz University Hospital (KAUH), Jeddah, Saudi Arabia.

## Materials and methods

Population and study design

This retrospective, cross-sectional study was carried out on 211 T2DM patients from both genders at KAUH in the year 2017, and the Research Ethics Committee of KAUH approved this study, approval No. 18-774. The data were collected by reviewing the electronic files of the patients.

Patient selection and biological measurements

Patients with an established diagnosis of T2DM were selected according to the criteria set by the American Diabetes Association in 2007. A patient’s smoking and alcohol history; their anthropometric measurements (weight, height, and BMI) and blood pressure; and their laboratory results, including HbA1c, transaminases (ALT, AST), TG, TC, LDL-C, and HDL-C levels, were noted. We included only those patients having T2DM. Patients suffering from type 1 diabetes mellitus; cirrhosis; or hepatitis A, B, or C, as well as alcoholics and patients taking medications that increase liver enzymes, were all excluded from the study.

Five milliliters of blood were taken after overnight fasting, and TG, TC, HDL-C, LDL-C, AST, ALT, HbA1c, and fasting blood sugar were measured. Lipid profile components were determined by using the cholesterol oxidase phenol 4-aminoantipyrine peroxidase (CHOD-PAP) method and kits on a Siemens Dimension Vista 1500 (Siemens Medical Solutions, Malvern, PA, USA). Blood sugar was measured using the glucose oxidase-peroxidase (GOD-POD) method.

ALT and AST were measured by clinical chemistry analyzer on the Siemens Dimension Vista 1500. The range for ALT was 12-78 U/L and AST was 15-37 U/L, according to KAUH laboratory normal limits. Values above these were taken as elevated.

Statistical analysis

The data were analyzed with SPSS 21 statistical software (IBM Corp., Armonk, NY, US), and quantitative variables are expressed as mean ± standard deviation. Smoking and hypertension are presented as frequencies and percentages, respectively. An independent t-test was used for a gender-wise comparison. ALT and AST association with different variables were calculated using a chi-square test. The odds ratio with a 95% confidence interval (CI) was computed for determining the association of any specific variable with increased levels of ALT and AST. p-value <0.5 was taken as significant.

## Results

The study included 211 T2DM patients with a mean age of 60 ± 13.43 years; 143 (67.8%) were female and 68 (32.2%) were male. The mean AST and ALT levels were 21.31 ± 14.38, and 32.71 ± 23.97, respectively. The mean HbA1c and BMI were 7.68 ± 1.8 and 30.69 ± 6.1, respectively. The participants’ baseline characteristics are shown in Table 1.

**Table 1:** The baseline characteristics and gender-wise comparison of the study subjects. BMI = Body mass index, HbA1c = Hemoglobin A1C, AST = Aspartate aminotransferase, ALT = Alanine aminotransferase, TC = Total cholesterol, HDL = High-density lipoprotein, LDL = Low-density lipoprotein, FBG = Fasting blood glucose

Variables	Total Mean±SD (N=211)	Female (n=143)	Male (n=68)	p-value
Age (Years)	60.00±13.43	58.34±13.3	63.48±13.08	0.009
BMI (Kg/m2)	30.69±6.1	31.67±6.5	28.62±4.4	<0.001
HgA1c (%)	07.68±1.8	07.88±1.8	07.24±1.5	<0.009
TC (mmol/L)	04.31±1.1	04.52±1.0	03.86±1.0	<0.001
HDL (mmol/L)	01.28±0.85	01.29±0.4	01.25±1.3	0.785
LDL (mmol/L)	02.63±0.92	02.81±0.9	02.27±0.8	<0.001
TG (mmol/L)	01.62±0.97	01.59±0.8	01.69±1.1	0.520
FBG (mmol/L)	08.13±2.9	08.51±3.1	07.35±2.4	0.005
AST U/L	21.31±14.38	20.13±12.3	22.60±16.3	0.14
ALT U/L	32.71±23.97	31.71±22.9	27.58±12.1	0.43

The gender-wise comparison showed that age, BMI, HbA1c, TC, LDL-C, and FBG were significantly different. No significant difference was observed between AST or ALT levels between the genders (Table 1).

The level of serum AST was elevated in 6.16% (10.3% in males and 4.2% in females) while elevated ALT levels were found in 7.58% (11.8% in males and 5.6% in females) (Table 2).

**Table 2:** Prevalence of elevated AST and ALT levels and odds ratio in type 2 diabetes mellitus patients for various risk factors. HbA1c = Hemoglobin A1C, AST = Aspartate aminotransferase, ALT = Alanine aminotransferase, TC = Total cholesterol, HDL = High-density lipoprotein, LDL = Low-density lipoprotein

Variables	Total	Increased AST activity				Increased ALT activity			
		n (%)	OR (95%)	X^2^	P-value	n (%)	OR (95%)	X^2^	P-value
Gender									
Male	68	7 (10.3)	2.62 (0.84–8.12)	2.96	0.85	8 (11.8)	2.25 (0.80–6.27)	2.50	0.11
Female	143	6 (4.2)	1			8 (5.6)	1		
Age groups									
24–45	27	1 (3.70%)	1			2 (10)	1		
46–65	101	9 (8.9)	2.59 (0.77–8.69)	2.53	0.11	6 (7.2)	1.09 (0.39–3.04)	0.03	0.85
>65	83	3 (3.6)	0.44 (0.11–1.65)	1.53	0.21	6 (7.2)	0.91 (0.32—2.6)	0.02	0.87
Smoking									
No	199	13 (6.5)	-	0.83	0.36	16 (8)	-	1.04	0.30
Yes	12	0 (0)	-			0 (0)	-		
HbA1c									
<7	91	7 (7.7)	0.63 (0.20–1.94)	0.64	0.42	9 (9.9)	0.56 (0.20–1.57)	1.21	0.27
>7	120	6 (5)	1			7 (5.8)	1		
TC									
<5.18	169	11 (6.5)	0.71 (0.15–3.37)	0.17	0.673	13 (7.7)	0.92 (0.25–3.39)	0.01	0.90
>5.18	42	2 (4.8)	1			3 (7.1)	1		
LDL									
<100	115	6 (5.2)	1.42 (0.464–4.40)	0.38	0.53	6 (5.2)	2.11 (0.73–6.04)	2.01	0.15
>100	96	7 (7.3)	1			10 (10.4)	1		
HDL									
<40	147	11 (7.5)	0.39 (0.08–1.85)	1.46	0.22	12 (8.2)	0.75 (0.23–2.42)	0.23	0.62
>40	64	2 (3.1)	1			4 (6.3)	1		
Triglycerides									
<150	151	9 (6)	1.12 (0.33–3.80)	0.13	0.84	9 (6)	2.08 (0.739–5.87)	1.99	0.16
>150	60	4 (6.7)	1			7 (11.7)	1		
Hypertension									
No	165	11 (6.7)	0.63 (0.13–2.97)	0.335	0.56	15 (9.1)	0.22 (0.02–1.72)	2.45	0.11
Yes	46	2 (4.3)				1 (2.2)	1		

The probability of elevated AST levels increased with increased age (OR = 2.59 for patients aged 46-65) and with male gender (OR = 2.65, CI: 0.84-8.12). Additionally, the probability of raised ALT levels increased with male gender (OR = 2.25, CI: 0.80-6.27), LDL-C (OR = 2.11, CI: 0.73-6.04), or TG (OR = 2.08, CI: 0.739-5.87) (Table 2).

No statistically noteworthy association was observed between the elevated levels of AST and ALT with gender, age, BMI, HbA1c, TC, TG, HDL-C, LDL-C, smoking, or hypertension (Table 2).

## Discussion

The findings from this study showed that the levels of serum AST and ALT were elevated in 6.16% and 7.58% of T2DM patients, respectively. Our studies are in accordance with several previous studies that also found serum transaminases were elevated in T2DM [7,10-11,18-19].

Our study indicates that the prevalence of elevated liver enzymes was gender-related, with elevated AST levels in 10.3% of males and 4.2% of females and elevated ALT levels in 11.8% of males and 5.6% of females. A similarly higher prevalence among males was reported from China by Chen et al. [11] and from Jordan by Judi et al. [16]. An Algerian study reported the prevalence of elevated ALT in T2DM patients was 15.9% in women and 10.9% in men [20].

The present study found no statistically significant association between the elevated levels of AST and ALT with gender, age, BMI, HbA1c, TC, TG, HDL-C, LDL-C, smoking, or hypertension, although a Jordanian study reported that male gender, younger age, higher waist circumference, and noninsulin usage were independent predictors of raised levels of liver transaminase [16].

Our results indicate the prevalence of elevated AST levels increased with increased age (OR = 2.59 for patients aged 46-65) and in the male gender (OR = 2.65, CI: 0.84-8.12). These results are in agreement with Gouri et al. [6] and Hermos et al. [21], who also reported elevated ALT levels with increasing age. Moreover, a study reported that male gender and elevated TG levels are independent predictors of ALT levels [6]. However, in our study, the odds ratio for AST and ALT elevation was only 0.44 (CI: (0.11-1.65)) and 0.91 (CI: (0.32-2.63)), respectively, among patients older than 65 years. We don’t have any particular explanation for this.

Elevated ALT values might be an important risk indicator for T2DM and metabolic syndrome (MS), and, in this context, it becomes imperative to identify the factors linked with elevated liver enzymes. These identified factors could assist in the prevention of T2DM and MS [7,22].

It is proposed that the elevated levels of ALT in diabetic patients and MS are chiefly due to fat accumulation in the liver [10,16]. Furthermore, elevated levels of ALT without any liver disease is usually taken as a surrogate marker of NAFLD [23]. The elevation of ALT could be because of impairment in insulin signaling rather than being purely due to hepatocyte injury [24].

A proposed pathophysiological mechanism is that elevated transaminases may indicate inflammation that weakens insulin signaling in the liver systemically [25-26]. Zhang et al. [27] stated that insulin resistance is caused by ALT regardless of gender, BMI, or age.

We found no significant difference according to BMI while another study reported a risk of increased ALT in diabetic patients with obesity [11]. Furthermore, the measurement of transaminases is more important in obese diabetic subjects according to one study that stated that the presence of T2DM, obesity, and elevated ALT, AST, WC are independent risk factors for fatty liver [18].

Another study mentioned that factors such as dietary habits, cultural influences, and awareness of health and body might play a role in the normalization of liver serum enzyme levels among the majority of people [20]. Our and other findings support the idea of implementing routine liver function test (LFT) monitoring in T2DM patients, as suggested by Gouri et al. [6].

There are a few limitations to our study. The first limitation is that it is a retrospective study, so direct patient histories could not be taken and we had to depend on existing electronic records. Second, the sample size is not large enough, and third, it is a single-center study and has no control group. Fourth, there is a lack of dietary history and other investigations such as abdominal sonography and did not discuss the presence of diabetic complications.

## Conclusions

Higher ALT and AST levels were found in T2DM patients. However, no statistically considerable link was observed between elevated levels of AST or ALT with gender, age, BMI, HbA1c, TG, TC, HDL-C, LDL-C, smoking, and hypertension. Future prospective longitudinal studies are recommended to find better results among Saudi diabetics as well as healthy subjects.
